# Commentary: Out-of-Body Experience during Awake Craniotomy

**DOI:** 10.3389/fnhum.2017.00417

**Published:** 2017-08-21

**Authors:** Estelle Nakul, Christophe Lopez

**Affiliations:** Centre National de la Recherhe Scientifique (LNIA, FR3C), Aix-Marseille Université Marseille, France

**Keywords:** embodiment, temporo-parietal junction (TPJ), out-of-body experience, multisensory integration, neurology, diffusion tensor imaging, electrocorticography (ECoG)

Out-of-body experience (OBE) is a rare phenomenon during which one has the feeling of being located outside of the physical boundaries of the body, along with the sensation of perceiving the world from an elevated perspective. OBE is of particular interest for studying self-consciousness, as it involves abnormal forms of self-location and visuo-spatial perspective.

OBE has been the subject of extensive investigations in parapsychology, neurology and cognitive neuroscience (Blackmore, 1982; Blanke, 2012). According to current neuroscientific models of embodiment, OBEs of neurological origin may arise from the conflict between sensory signals indicating how the body and environment are oriented. The abnormal integration of visual and vestibular signals with somatosensory (tactile and proprioceptive) signals can explain disembodied self-location, sensations of lightness and floating of the self, complex visual illusions (disembodied viewpoint, autoscopy) and distortions of the body schema often reported during OBE (Blanke et al., 2004; Lopez et al., 2008; Lopez and Elzière, 2017). In addition, abnormal multisensory integration has recently been reported in neurologically normal individuals who experienced an OBE (Braithwaite et al., 2017). Regarding the neural basis of OBE, the temporo-parietal junction (TPJ) has repeatedly been shown to play a crucial role in anchoring the self to the body, since stimulation and lesion to the TPJ can evoke a disembodied experience (Simeon et al., 2000; Blanke et al., 2002; De Ridder et al., 2007; Ionta et al., 2011). A recent study of OBE provides new evidence of multisensory misintegration at the TPJ and extends these findings.

Bos et al. (2016) report the case of a 50-year-old woman who underwent awake craniotomy for surgical resection of an oligodendroglioma grade II, located at the left angular and supramarginal gyrus (Figure 1A). During resection of the tumor, electrical stimulation of the peritumoral subcortical tissue at the left TPJ first evoked the feeling that the patient's right leg was “*drawn toward the opposite wall of the operating theater”*, suggesting that TPJ stimulation distorted her body schema. During subsequent stimulations of the same area, she reported three full-blown OBEs, in which she felt “*as if she was floating just below the ceiling and saw her own body lying on the operating table”* (i.e., autoscopy). She never reported OBE before or after surgery.

**Figure 1:**
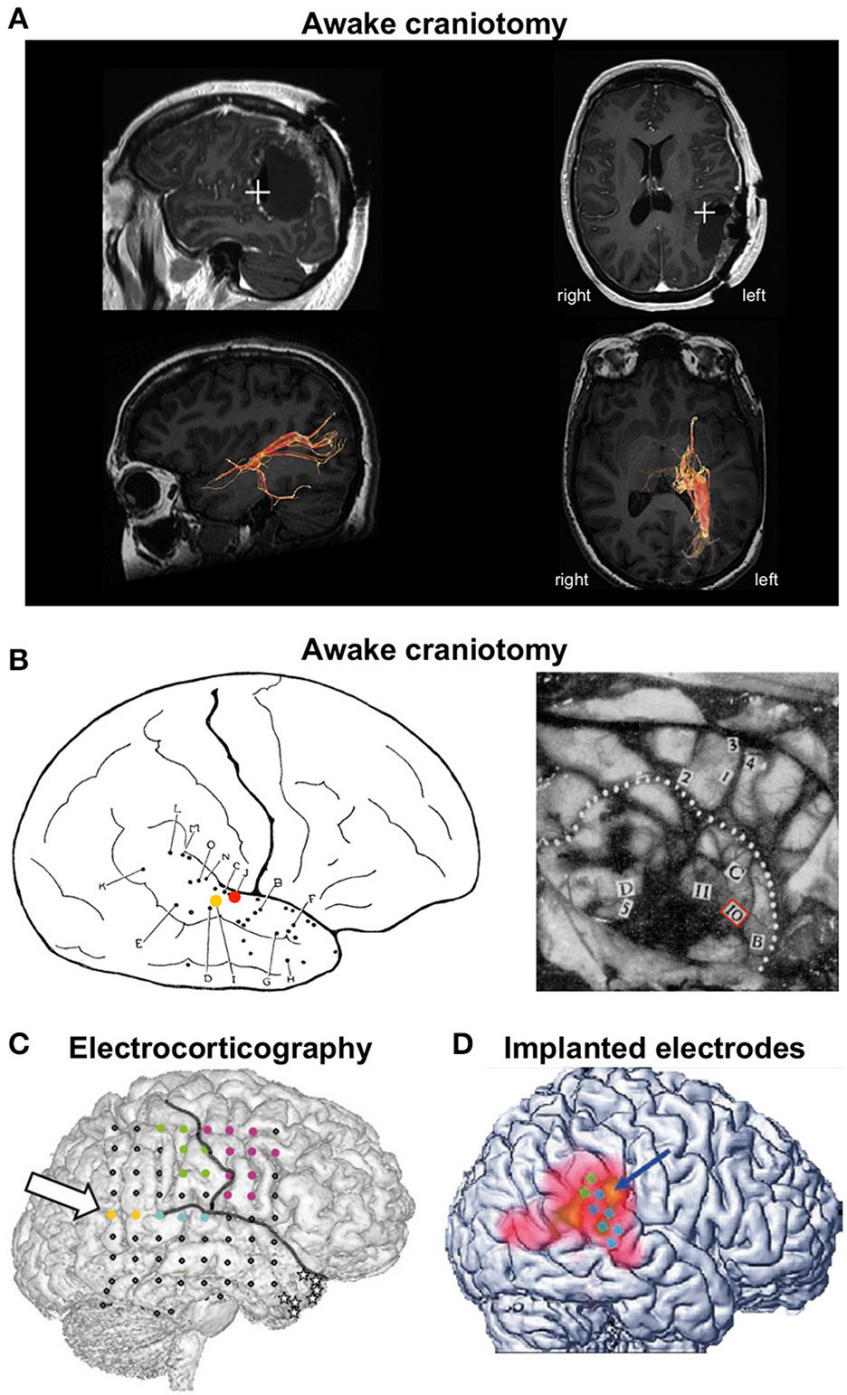
Cases of out-of-body experience during electrical brain stimulation. **(A)** OBE evoked during awake craniotomy in a 50-year-old female patient for surgical resection of an oligodendroglioma located at the left angular and supramarginal gyrus. Stimulation at the left TPJ evoked full-blown OBEs “*as if she was floating just below the ceiling and saw her own body lying on the operating table.”* Top row: postoperative magnetic resonance imaging showing the location of the stimulation (white cross) that evoked OBEs. Bottom row: diffusion tensor imaging tractography of the posterior thalamic radiations. The stimulated region was close to fibers running from the posterior thalamus to the occipital lobe. Reproduced from Bos et al. (2016) with permission from Elsevier. **(B)** Seminal descriptions of OBEs during awake craniotomy. The left panel illustrates a female patient (case G.A.) undergoing surgery for epilepsy treatment. During stimulation at point I (yellow dot) G.A. reported “*I feel queer, as though I were floating away.”* Stimulation at point J (red dot) evoked the feeling that “*I have a queer sensation as if I am not here”* and “*As though I were half and half here.”* Reproduced from Penfield (1947) with permission from The Royal Society. The right panel illustrates case V.F., a 33-year-old male patient who suffered from seizures due to an atrophic discharging lesion deep in the right temporal region near the insula. The right temporal lobe was exposed for temporal excision. After stimulation at point 10, V.F. exclaimed “*Oh God! I am leaving my body.”* Reproduced from Penfield (1955) with permission from the Royal College of Psychiatrists. **(C)** OBE evoked during electrocorticography in a 43-year-old female patient with right temporal lobe epilepsy. Electrical stimulation at low intensity between the yellow points at the right angular gyrus (arrow) induced vestibular sensations and body schema illusions. Stimulation at higher intensity evoked full-blown OBEs with disembodied self-location and autoscopy: “*I see myself lying in bed, from above, but I only see my legs and lower trunk.”* Reproduced from Blanke et al. (2002) with permission from Nature Publishing Group. **(D)** OBE evoked in a 63-year-old male patient who had electrodes implanted in his right TPJ for suppression of intractable tinnitus. Stimulation of the TPJ induced perception of disembodiment, as if he were located about 50 cm behind his body and off to the left. There was no autoscopy and the visual environment was experienced from a body-centered perspective. Reproduced from De Ridder et al. (2007) with permission from the Massachusetts Medical Society.

This case is remarkable as it is the first to report OBE during an *awake craniotomy* since seminal observations by the Canadian neurosurgeon Wilder Penfield (Penfield, 1947, 1955). We found only five published cases of OBE in the entire history of brain stimulation: three during awake craniotomy, one during electrocorticography, and one with chronically implanted electrodes (Figure 1). OBE seems extremely rare when compared to the large range of perceptual illusions reported during electrical brain stimulation (reviewed in Selimbeyoglu and Parvizi, 2010). To date, there is no answer as to why OBE is so rarely induced by electrical brain stimulation, considering that 5–10% of neurologically normal individuals have had an OBE (Blanke and Dieguez, 2009). This indicates that the neural underpinnings of the perceived anchoring of the self to the body are robust. Interestingly, all published cases of OBE following brain stimulation involved the TPJ. This is consistent with a large body of evidence showing that the TPJ is a core region for self-processing and perspective taking (Decety and Lamm, 2007; Blanke, 2012; Wang et al., 2016). But why did these five patients report OBEs and no other patients stimulated at the TPJ? Do they belong to an OBE-prone subpopulation? These questions have not been addressed. We believe it will be important to determine the precipitating factors of OBE for a better understanding of the neurophysiological and psychological mechanisms of embodiment. We propose that future clinical studies measure depersonalization-derealization, along with multisensory integration, using paradigms designed to evoke OBE–like experiences (Ehrsson, 2007; Lenggenhager et al., 2007).

Another novel aspect of the case report by Bos and colleagues is that it is the only case of OBE evoked during electrical stimulation of *subcortical tracts*. The originality of the present study also lies in joining diffusion tensor imaging (DTI) tractography to the study of OBE. DTI revealed that the stimulated brain region was close to fibers running from the posterior thalamus to the occipital lobe (Figure 1A). This finding is important as it suggests that, in addition to involving the TPJ, the electrical stimulation “activates” a network of brain regions accounting for the complex phenomenal experience of OBEs. Both the TPJ and posterolateral thalamus have been involved in the sense of self-orientation and uprightness (Kheradmand et al., 2015; Baier et al., 2016; Kirsch et al., 2017) and the medial occipital cortex has been involved in autoscopic hallucinations (Jonas et al., 2014). A PET study during OBEs evoked by cortical electrical stimulation revealed activation beyond the TPJ, in the precuneus and the posterior thalamus (De Ridder et al., 2007). The exact contributions of these different brain areas to OBE are unknown. Future studies should identify the cerebral regions that, together with TPJ, support the various facets of OBE and describe the connectivity between those regions. Accordingly, DTI studies in patients and neurologically normal individuals who have had an OBE should be conducted to identify the brain regions involved in the various perceptual contents of OBEs and OBE–like experiences. Special attention should be given to the relation between the exact phenomenological experience (e.g., do individuals report seeing their own body, controlling the motion of their disembodied self, experience vestibular sensations and distortions of their body schema?) and studies of brain anatomy and connectivity.

Interestingly, OBEs are also a common feature of near-death experiences. Studies of disembodied experiences during near-death experiences focusing on changes in cortical anatomy and surface electroencephalography have proven controversial (Blanke and Dieguez, 2009; Agrillo, 2011). Results from the report by Bos and colleagues, as well as other studies that evoked illusory self-motion during subcortical tract stimulation (Spena et al., 2006), motivate investigations into the contribution of subcortical tracts to complex bodily perceptions and self-consciousness.

The present case is also the only description of OBE during stimulation of the left TPJ. The left TPJ has been involved in complex bodily illusions, such as the induction of an illusory shadow person (Arzy et al., 2006), but less frequently in disembodied experiences (Blanke et al., 2004). Although a dominance of the right TPJ for OBE is consistent with a right hemispheric dominance for vestibular (self-motion) information processing (Dieterich et al., 2003) and perspective taking (Wang et al., 2016), more should be known about the respective role of right and left TPJ in OBEs.

In conclusion, cases of OBE evoked by intracranial stimulation and focal brain damage should be reported systematically in the literature for a better understanding of the neural bases of embodiment. Future studies combining DTI, resting-state functional connectivity and measures of cortical thickness in neurological patients and neurologically normal individual with OBE will foster significant advances in the neuroscience of OBE, similar to studies of xenomelia, schizophrenia and depersonalization (Simeon et al., 2000; Kubicki et al., 2007; Hilti et al., 2013). Finally, future work should pay special attention to the methods and parameters of stimulation, the issue of hemispheric laterality and a more systematic study of the phenomenology of OBE.

## Author contributions

All authors listed have made a substantial, direct and intellectual contribution to the work, and approved it for publication.

### Conflict of interest statement

The authors declare that the research was conducted in the absence of any commercial or financial relationships that could be construed as a potential conflict of interest.
